# IQWiG-Evidenzbericht zum Thema psychische Folgen eines Schwangerschaftsabbruchs

**DOI:** 10.1007/s00103-024-03993-4

**Published:** 2024-12-12

**Authors:** David Endres, Julia Gowik, Andrea Tasar

**Affiliations:** Ressort Versorgung & Leitlinien, Institut für Qualität und Wirtschaftlichkeit im Gesundheitswesen, Siegburger Str. 237, 50679 Köln, Deutschland

**Keywords:** Psychische Gesundheit, Schwangerschaft, Erstes Trimenon, Leitlinien, Systematische Übersicht, Mental health, Pregnancy, First trimester, Guidelines, Systematic review

## Abstract

Das Institut für Qualität und Wirtschaftlichkeit im Gesundheitswesen (IQWiG) wurde vom Bundesministerium für Gesundheit beauftragt, bei der Weiterentwicklung der S2k-Leitlinie „Schwangerschaftsabbruch im ersten Trimenon“ zu einer S3-Leitlinie zu unterstützen. Dazu wurden von der zuständigen Leitliniengruppe Fragestellungen formuliert, die in Evidenzberichten des IQWiG beantwortet wurden. Für eine dieser Fragestellungen sollte die Evidenz bezüglich der psychischen Folgen eines Schwangerschaftsabbruchs im ersten Trimenon im Vergleich zu keinem Schwangerschaftsabbruch im ersten Trimenon bei Schwangeren mit dem Wunsch nach einem Schwangerschaftsabbruch dargestellt werden. In einer systematischen Recherche wurde eine relevante prospektiv vergleichende Kohortenstudie identifiziert, die Ergebnisse zu den Endpunkten klinisch diagnostizierte Depressionen und klinisch diagnostizierte Angststörungen berichtete. Die Evidenz wurde auf Grundlage der methodischen Vorgaben von „Grading of Recommendations Assessment, Development and Evaluation“ (GRADE), konkretisiert durch die IQWiG-Methodik, beurteilt. Es konnten keine signifikanten Unterschiede zwischen den Gruppen zu den genannten Endpunkten festgestellt werden. Die Vertrauenswürdigkeit der Evidenz wurde als sehr niedrig eingestuft, Abwertungen ergaben sich durch Studienlimitationen und fehlende Genauigkeit der Effekte. Prospektive vergleichende Kohortenstudien, die sich der untersuchten Fragestellung nähern, sollten unter anderem eine adäquate Kontrolle für relevante Störgrößen, eine ausreichende Anzahl an Teilnehmerinnen sowie eine sorgfältig geplante Erhebung relevanter Endpunkte aufweisen.

## Abstract (en)

The Institute for Quality and Efficiency in Health Care (IQWiG) was commissioned by the Federal Ministry of Health to provide support for the further development of the S2k guideline on first-trimester abortion into an S3 guideline. To this end, the responsible guideline group formulated research questions that were answered in IQWiG evidence reports. One of the questions to be addressed was the evidence regarding the psychological consequences of an abortion in the first trimester compared to no abortion in the first trimester in pregnant women who wish to have an abortion. A systematic search identified one relevant prospective comparative cohort study that reported results on the outcomes of clinically diagnosed depression and clinically diagnosed anxiety disorders. The evidence was evaluated in accordance with the methodological requirements of the Grading of Recommendations Assessment, Development and Evaluation (GRADE) working group, as specified by the IQWiG methods. No significant differences were observed between the study groups with regard to the outcomes mentioned. The certainty of the evidence was rated as very low and downgraded due to study limitations and imprecision of the effects. Prospective comparative cohort studies that approach the research question under investigation should, inter alia, have adequate control for relevant confounders, a sufficient number of participants, and a carefully planned collection of data on relevant outcomes.

## Einleitung

Das *Institut für Qualität und Wirtschaftlichkeit im Gesundheitswesen* (IQWiG) veröffentlichte im Juni 2023 eine Evidenzrecherche zur Unterstützung der Weiterentwicklung der S2k-Leitlinie „Schwangerschaftsabbruch im ersten Trimenon“ zu einer S3-Leitlinie [1]. Diese Evidenzrecherche wurde gemäß SGB V (§§ 139a Abs. 3 Nr. 3, 139b Abs. 6) vom *Bundesministerium für Gesundheit* in Auftrag gegeben und umfasste 8 Fragestellungen zu unterschiedlichen Aspekten der Versorgung von Schwangeren, die einen Schwangerschaftsabbruch durchführen lassen. Die Fragestellungen wurden von der zuständigen Leitliniengruppe formuliert und in Zusammenarbeit mit der *Arbeitsgemeinschaft der Wissenschaftlichen Medizinischen Fachgesellschaften e.* *V.* und dem IQWiG konkretisiert. Pro Fragestellung wurde ein Evidenzbericht erstellt. Jeder Evidenzbericht beinhaltet die zur Fragestellung aufbereitete systematische Recherche und die Bewertung der Vertrauenswürdigkeit der zur Verfügung stehenden Evidenz, die in Evidenzprofilen dargestellt wird. Evidenzberichte enthalten keine Empfehlungen für oder gegen z. B. eine Intervention oder ein diagnostisches Testverfahren. Sie dienen als Evidenzgrundlage im Rahmen der Empfehlungsgenerierung durch die Leitliniengruppe.

In der aktuellen S2k-Leitlinie Schwangerschaftsabbruch im ersten Trimenon wird die Frage betrachtet, ob Schwangerschaftsabbrüche negative Auswirkungen auf die psychische Gesundheit haben. Diese Frage wurde in vielen Studien untersucht, wobei die Beurteilung der Daten aufgrund methodischer Probleme der Forschung zu diesem Thema, methodischer Mängel der Studien und aufgrund ethischer und politischer Aspekte kontrovers bleibt. Auf Basis systematischer Übersichten und Studien bestehen keine Unterschiede im Auftreten von späteren psychischen Problemen oder Erkrankungen zwischen der Austragung oder dem Abbruch einer ungewollten Schwangerschaft. Dennoch bleibt unklar, welche Bedeutung psychische Probleme in der Nachbetreuung von Frauen nach Schwangerschaftsabbrüchen haben [2]. Um einen Beitrag zur Beurteilung der kontrovers diskutierten Datenlage zum Thema der psychischen Gesundheit von Frauen nach einem Schwangerschaftsabbruch zu leisten, wurde das IQWiG mit einer unabhängigen Aufbereitung der Evidenz zu möglichen psychischen Folgen eines Schwangerschaftsabbruchs beauftragt. Im vorliegenden Beitrag werden die wesentlichen Ergebnisse des IQWiG-Evidenzberichts beschrieben und eingeordnet sowie Implikationen für das Forschungsfeld herausgestellt.

## Fragestellung des Evidenzberichts

Im Evidenzbericht des IQWiG wurde die Evidenz bezüglich der psychischen Folgen eines Schwangerschaftsabbruchs bis zum Ende der *Schwangerschaftswoche* (SSW) 14 nach Beginn der letzten Menstruation (innerhalb des ersten Trimenons) im Vergleich zu keinem Schwangerschaftsabbruch bis zum Ende der SSW 14 nach Eintritt der letzten Menstruation bei Schwangeren mit Wunsch nach einem Schwangerschaftsabbruch dargestellt. Kein Schwangerschaftsabbruch bis zum Ende der SSW 14 nach Eintritt der letzten Menstruation umfasste hierbei entweder

- eine Austragung der Schwangerschaft,
- einen Schwangerschaftsabbruch nach dem ersten Trimenon oder
- eine Fehlgeburt nach dem ersten Trimenon.

Gemäß Festlegung der Leitliniengruppe sollten folgende Endpunkte betrachtet werden:

- akute Belastungsreaktionen,
- posttraumatische Belastungsstörungen,
- andere psychiatrische Erkrankungen,
- Angststörungen,
- Konsumstörungen (Alkohol und andere Drogen),
- Suizidversuche,
- nichtsuizidales selbstverletzendes Verhalten.

Für die genannten psychischen und psychiatrischen Erkrankungen und Störungen sollte eine Diagnose nach ICD (International Statistical Classification of Diseases and Related Health Problems) oder DSM (Diagnostic and Statistical Manual of Mental Disorders) vorliegen [1].

## Methodik des Evidenzberichts

Für die Bearbeitung der Fragestellung erfolgten eine systematische Literaturrecherche, die Selektion relevanter Studien sowie die Extraktion der Studiencharakteristika und Daten zu vorhandenen Endpunkten. Die Erstellung der Evidenzprofile erfolgt auf Grundlage der methodischen Vorgaben von „Grading of Recommendations Assessment, Development and Evaluation“ (GRADE; [3]) und wurde durch die IQWiG-Methodik [4] konkretisiert. Für jeden Endpunkt und Vergleich wurde eine studienübergreifende Aussage zur Vertrauenswürdigkeit der Evidenz getroffen. Hierzu erfolgte eine Prüfung hinsichtlich einer möglichen Abwertung der Vertrauenswürdigkeit der Evidenz durch Studienlimitationen, des Risikos eines Publikationsbias, der fehlenden Genauigkeit und Inkonsistenz der Effekte sowie der Übertragbarkeit. Auch mögliche Aspekte zur Aufwertung der Vertrauenswürdigkeit der Evidenz wurden geprüft, beispielsweise das Vorliegen sehr großer Effekte. Die Vertrauenswürdigkeit der Evidenz wurde entsprechend den 4 Stufen der GRADE-Guideline in hoch, moderat, niedrig und sehr niedrig eingeteilt [5–7]. Die vollständige methodische Vorgehensweise ist dem Evidenzbericht zu entnehmen [1].

Nachfolgend wird ein Überblick über die wesentlichen Ergebnisse des Evidenzberichts gegeben, gefolgt von einer Einordnung der mit der Fragestellung verbundenen Evidenzlage sowie einer Ableitung möglicher methodischer Implikationen für das Forschungsfeld.

## Ergebnisse des Evidenzberichts

Im Rahmen der systematischen Literaturrecherche wurden 891 Referenzen aus bibliografischen Datenbanken und Studienregistern gesichtet. Die vollständige Suchstrategie ist im Evidenzbericht abgebildet [1]. Nach dem Screening der Titel und Abstracts wurden 86 Volltexte geprüft. Insgesamt wurde 1 relevante Studie identifiziert, welche alle vorgegebenen Einschlusskriterien (Tab. 1) erfüllte.

**Tab. 1:** Übersicht über die Kriterien für den Studieneinschluss [1]

Einschlusskriterien	
E1	Population: Schwangere mit Wunsch nach einem Schwangerschaftsabbruch (z. B.: werden in einer medizinischen Einrichtung und/oder Beratungsstelle vorstellig, in der operative oder medikamentöse Schwangerschaftsabbrüche durchgeführt werden)
E2	Studiengruppe der Frauen, die einen Schwangerschaftsabbruch bis zur SSW 14 + 0 p. m. hatten
E3	Studiengruppe der Frauen, die keinen Schwangerschaftsabbruch bis zur SSW 14 + 0 p. m. aus verschiedenen Gründen hatten
E4	Die Darstellung der Endpunkte erfolgt unter Berücksichtigung folgender Aspekte im weiteren Verlauf: eine Austragung der Schwangerschaft, ein späterer Schwangerschaftsabbruch (> SSW 14 + 0 p. m.) oder eine spätere Fehlgeburt
	Kritische Endpunkte: akute Belastungsreaktion, posttraumatische Belastungsstörung, andere psychiatrische Erkrankung (als Gesamtrate)
	Wichtige Endpunkte: Angststörung, Konsumstörung (Alkohol und andere Drogen), Suizidversuch, nichtsuizidales selbstverletzendes Verhalten
	Bei mehr als 7 operationalisierten Endpunkten sind kritische Endpunkte den wichtigen Endpunkten vorzuziehen
E5	Studientypen: RCTs und ggf. nicht randomisierte vergleichende Studien (schrittweiser Einschluss von niedrigeren Evidenzstufen bei ungenügender Zahl und/oder Qualität in der nächst höheren Evidenzstufe: quasirandomisierte kontrollierte Studien, prospektive vergleichende Kohortenstudien, retrospektive vergleichende Kohortenstudien mit zeitlich paralleler Kontrollgruppe, retrospektive vergleichende Kohortenstudien mit nicht zeitlich paralleler Kontrollgruppe; zu diesem Vorgehen gab es eine Spezifizierung im Projektverlauf, siehe Evidenzbericht Abschnitt 3.2 [1])
E6	Nachbeobachtungsdauer: bis zu 10 Jahre
E7	Publikationssprache: Deutsch oder Englisch
E8	Vollpublikation verfügbar^a^

^a^ Als Vollpublikation gilt in diesem Zusammenhang auch ein Studienbericht gemäß ICH E3 [9] oder ein Bericht über die Studie, der den Kriterien des CONSORT- [10], TREND- [11] oder STROBE-Statements [12] genügt und eine Bewertung der Studie ermöglicht, sofern die in diesen Dokumenten enthaltenen Informationen zur Studienmethodik und zu den Studienergebnissen nicht vertraulich sind

*p.* *m.* post menstruationem (nach der letzten Menstruation), *CONSORT* Consolidated Standards of Reporting Trials, *ICH* International Council for Harmonisation of Technical Requirements for Pharmaceuticals for Human Use, *RCTs* randomisierte kontrollierte Studien, *SSW* Schwangerschaftswoche, *STROBE* Strengthening the Reporting of Observational Studies in Epidemiology, *TREND* Transparent Reporting of Evaluations with Nonrandomized Designs

Bei der eingeschlossenen Studie handelt es sich um die aus den USA stammende Turnaway-Studie, eine prospektiv vergleichende Kohortenstudie mit zeitlich parallelen Studiengruppen [8]. In der Studie wurden die physische und psychische Gesundheit sowie sozioökonomische Faktoren bei Schwangeren mit Wunsch nach einem Schwangerschaftsabbruch untersucht. Unterschieden wurden Frauen, die einen Schwangerschaftsabbruch durchführen ließen, von denen, denen ein Abbruch verwehrt wurde. Für den Evidenzbericht wurde die Zwischenauswertung nach 3 Jahren der insgesamt 5 Jahre andauernden Studie herangezogen. In dieser wurden Daten zu 877 Frauen berichtet, die eine Woche nach dem jeweiligen Ereignis (Durchführung oder Verwehrung des Schwangerschaftsabbruchs) und anschließend halbjährlich über 3 Jahre telefonisch unter anderem zu einer neu aufgetretenen klinisch diagnostizierten Depression oder einer klinisch diagnostizierten Angststörung befragt wurden.

Zur Beantwortung der Fragestellung des Evidenzberichts wurden alle 4 Studiengruppen der Zwischenauswertung herangezogen (Tab. 2):

- Studiengruppe 1: Schwangere, die einen Schwangerschaftsabbruch im ersten Trimenon durchführen ließen,
- Studiengruppe 2: Schwangere, deren Wunsch nach einem Schwangerschaftsabbruch abgelehnt wurde und die in Folge die Schwangerschaft austrugen,
- Studiengruppe 3: Schwangere, deren Wunsch nach einem Schwangerschaftsabbruch initial abgelehnt wurde und die im weiteren Verlauf eine Fehlgeburt erlitten oder die in einer anderen, nicht an der Studie beteiligten Einrichtung den Schwangerschaftsabbruch (nach dem ersten Trimenon) durchführen ließen,
- Studiengruppe 4: Schwangere, die einen Schwangerschaftsabbruch nach dem ersten Trimenon durchführen ließen (keine Ablehnung des Wunsches nach einem Abbruch).

**Tab. 2:** Charakterisierung der Studienpopulation innerhalb der Turnaway-Studie [1]

Studie, Gruppe	Studiengruppe 1^a^	Studiengruppe 2^b^	Studiengruppe 3^c^	Studiengruppe 4^d^
Biggs et al. (2015) (Turnaway-Studie)				
*N*	254	160	50	413
Alter (Jahre), MW (SD)	25,9 (5,7)	23,4 (5,5)	24,4 (6,2)	24,9 (5,9)
Gestationsalter (Wochen), MW (SD)	7,8 (2,4)	23,4 (3,4)	19,2 (4,0)	19,9 (4,1)
Keine vorherige Geburt, *n* (%)	97 (38)	75 (47)	20 (40)	140 (34)
Erlebter Kindesmissbrauch oder Vernachlässigung, *n* (%)	70 (28)	41 (26)	7 (14)	108 (26)
Unerlaubter Drogenkonsum vor der Schwangerschaft, *n* (%)	45 (18)	22 (14)	4 (8)	52 (13)
Problematischer Alkoholkonsum vor der Schwangerschaft, *n* (%)	18 (7)	11 (7)	5 (10)	18 (4)
Diagnostizierte Depressionen, *n* (%)	63 (25)	25 (16)	13 (26)	77 (19)
Diagnostizierte Angststörung, *n* (%)	41 (16)	19 (12)	9 (18)	62 (15)

^a^ Schwangerschaftsabbruch innerhalb des ersten Trimenons

^b^ Austragung der Schwangerschaft nach abgelehntem Wunsch eines Schwangerschaftsabbruchs

^c^ Fehlgeburt oder Schwangerschaftsabbruch nach dem ersten Trimenon nach initial abgelehntem Wunsch eines Schwangerschaftsabbruchs

^d^ Schwangerschaftsabbruch nach dem ersten Trimenon (keine Ablehnung des Wunsches nach einem Abbruch)

*MW* Mittelwert, *N* Anzahl eingeschlossener Frauen, *SD* Standardabweichung

Die Frauen in den Studiengruppen wiesen Unterschiede im Vorliegen von psychischen Vorerkrankungen oder psychischen Störungen auf. Für die Ergebnisse wurde in der Studie eine Adjustierung hinsichtlich dieser und weiterer soziokultureller Einflussfaktoren vorgenommen.

Für die in der Fragestellung definierten Vergleiche der Studiengruppen lagen Daten zu den Endpunkten *andere psychiatrische Erkrankungen* und *Angststörungen *vor. Unter dem Endpunkt *andere psychiatrische Erkrankungen *wurden die in der Studie berichteten neu aufgetretenen klinisch diagnostizierten Depressionen und unter dem Endpunkt *Angststörungen* die neu aufgetretenen klinisch diagnostizierten Angststörungen jeweils über einen Zeitraum von 3 Jahren erfasst.

Sowohl für *klinisch diagnostizierte Depressionen* als auch für *klinisch diagnostizierte Angststörungen* zeigten sich für alle Vergleiche der Studiengruppen keine statistisch signifikanten Unterschiede. Das Vertrauen in die Effektschätzungen wurde jeweils als sehr gering beurteilt (sehr niedrige Vertrauenswürdigkeit der Evidenz). Eine sehr niedrige Vertrauenswürdigkeit der Evidenz bedeutet, dass der wahre Effekt durchaus relevant sehr verschieden von der Effektschätzung sein kann [5–7]. Die Abwertungen der Vertrauenswürdigkeit der Evidenz ergaben sich durch schwerwiegende Studienlimitationen und schwerwiegende bzw. sehr schwerwiegende fehlende Genauigkeit der Effekte (Tab. 3 und 4). Bei der Bewertung der fehlenden Genauigkeit, d. h. der Präzision der Effektschätzungen, wurden die Lage und Breite des 95 %-Konfidenzintervalls der jeweiligen Effektschätzung beurteilt.

**Tab. 3:** Evidenzprofil für den Vergleich Schwangerschaftsabbruch bis zur SSW 14 + 0 p. m. vs. kein Schwangerschaftsabbruch bis zur SSW 14 + 0 p. m. aus verschiedenen Gründen für den kritischen Endpunkt „andere psychiatrische Erkrankung“ [1]

Faktoren der Qualität der Evidenz							Anzahl der Frauen mit Ereignis/Anzahl der Frauen			Basisrisiko in %^a^	Effekt		Qualität der Evidenz^b^
Studiendesign; *N*	Studienlimitationen^c^	Inkonsistenz	Indirektheit	Publikationsbias	Fehlende Genauigkeit	Andere Faktoren	(I)		(C)		OR^d^	RD in %‑Punkten	
											[95 %-KI]	[95 %-KI]	
**Studiengruppe 1 vs. 2: Schwangerschaftsabbruch bis zur SSW 14 + 0 p. m. versus Austragung der Schwangerschaft nach abgelehntem Wunsch eines Schwangerschaftsabbruchs**													
**Klinisch diagnostizierte Depression (von den Frauen berichtet)**													
Interpretation der Effektschätzung: Ein OR größer als 1 bzw. eine positive Effektschätzung bedeutet eine geringere Chance für die Entwicklung einer Depression in der Studiengruppe Schwangerschaftsabbruch bis zur SSW 14 + 0 p. m.													
Non-RCT; 1 [8]	Schwerwiegend^e^	Nicht zutreffend^f^	Nicht schwerwiegend	Unentdeckt	Schwerwiegend^g^	Keine	16/129		11/87	13	1,4	4	Sehr niedrig
											[0,62; 3,17]	[−4; 19]	
**Studiengruppe 1 vs. 3: Schwangerschaftsabbruch bis zur SSW 14** **+** **0 p.** **m. versus Fehlgeburt oder Schwangerschaftsabbruch nach SSW 14** **+** **0 p.** **m. nach initial abgelehntem Wunsch eines Schwangerschaftsabbruchs**													
**Klinisch diagnostizierte Depression (von den Frauen berichtet)**													
Interpretation der Effektschätzung: Ein OR größer als 1 bzw. eine positive Effektschätzung bedeutet eine geringere Chance für die Entwicklung einer Depression in der Studiengruppe Schwangerschaftsabbruch bis zur SSW 14 + 0 p. m.													
Non-RCT; 1 [8]	Schwerwiegend^e^	Nicht zutreffend^f^	Nicht schwerwiegend	Unentdeckt	Sehr schwerwiegend^h^	Keine	16/129		4/23	17	2,16	13	Sehr niedrig
											[0,44; 10,53]	[−9; 52]	
**Studiengruppe 1 vs. 4: Schwangerschaftsabbruch bis zur SSW 14 + 0 p. m. versus Schwangerschaftsabbruch nach SSW 14 + 0 p. m. (keine Ablehnung des Wunsches nach einem Abbruch)**													
**Klinisch diagnostizierte Depression (von den Frauen berichtet)**													
Interpretation der Effektschätzung: Ein OR größer als 1 bzw. eine positive Effektschätzung bedeutet eine geringere Chance für die Entwicklung einer Depression in der Studiengruppe Schwangerschaftsabbruch bis zur SSW 14 + 0 p. m.													
Non-RCT; 1 [8]	Schwerwiegend^e^	Nicht zutreffend^f^	Nicht schwerwiegend	Unentdeckt	Nicht schwerwiegend	Keine	16/129		27/220	12	1,1	1	Sehr niedrig
											[0,64; 1,92]	[−4; 9]	

^a^ Basisrisiko geschätzt durch (medianes) Risiko der Vergleichsgruppen der eingeschlossenen Studie (der Begriff „Risiko“ bezieht sich hier sowohl auf Endpunkte, deren Ereignisse sich für die Frauen negativ, als auch auf solche, deren Ereignisse sich positiv auswirken)

^b^ Unter Qualität der Evidenz wird die studienübergreifende endpunktbezogene Qualität bzw. Vertrauenswürdigkeit der Evidenz verstanden

^c^ Die Bewertung der Studienlimitationen pro Studie pro Endpunkt ist dem Evidenzbericht [2] zu entnehmen

^d^ Adjustiert für Ethnie, Alter, Parität, Bildung, Erwerbstätigkeit, Krankenversicherungsstatus, Drogen und Alkoholprobleme vor der Schwangerschaft, frühere Diagnose einer Angststörung, früherer Kindesmissbrauch oder Vernachlässigung

^e^ Die Vollständigkeit der Daten (Anteil der Frauen in der Auswertung mit fehlenden Werten zwischen ca. 10–30 %) und die Berücksichtigung prognostisch relevanter Faktoren (fehlende Adjustierung für „frühere Schwangerschaftsabbrüche“) waren nicht adäquat

^f^ Der Faktor inkonsistente (heterogene) Effekte kam für diesen Evidenzbericht nicht zur Anwendung, da jeweils nur 1 Studie pro Endpunkt vorlag

^g^ Das 95 %-KI des OR überdeckt 1 und 2. Somit können weder Effekte zuungunsten noch mittelgroße Effekte zugunsten der Studiengruppe 1 (Schwangerschaftsabbruch bis zur SSW 14 + 0 p. m.) ausgeschlossen werden

^h^ Das 95 %-KI des OR überdeckt 0,5 und 2. Somit können weder mittelgroße Effekte zuungunsten noch mittelgroße Effekte zugunsten der Studiengruppe 1 (Schwangerschaftsabbruch bis zur SSW 14 + 0 p. m.) ausgeschlossen werden

*C* kein Schwangerschaftsabbruch bis zur SSW 14 + 0 p. m., *I* Schwangerschaftsabbruch bis zur SSW 14 + 0 p. m., *KI* Konfidenzintervall, *N* Anzahl der Studien, *Non-RCT* nicht randomisierte kontrollierte Studie, *OR* Odds Ratio, *p.* *m.* post menstruationem, *RD* absolute Risikodifferenz, *SSW* Schwangerschaftswoche

**Tab. 4:** Evidenzprofil für den Vergleich Schwangerschaftsabbruch bis zur SSW 14 + 0 p. m. vs. kein Schwangerschaftsabbruch bis zur SSW 14 + 0 p. m. aus verschiedenen Gründen für den wichtigen Endpunkt „Angststörung“ [1]

Faktoren der Qualität der Evidenz							Anzahl der Frauen mit Ereignis/Anzahl der Frauen		Basisrisiko in %^a^	Effekt		Qualität der Evidenz^b^
Studiendesign; *N*	Studienlimitationen^c^	Inkonsistenz	Indirektheit	Publikationsbias	Fehlende Genauigkeit	Andere Faktoren	(I)	(C)		OR^d^	RD in %‑Punkten	
										[95 %-KI]	[95 %-KI]	
**Studiengruppe 1 vs. 2: Schwangerschaftsabbruch bis zur SSW 14** **+** **0 p.** **m. versus Austragung der Schwangerschaft nach abgelehntem Wunsch eines Schwangerschaftsabbruchs**												
**Klinisch diagnostizierte Angststörung (von den Frauen berichtet)**												
Interpretation der Effektschätzung: Ein OR größer als 1 bzw. eine positive Effektschätzung bedeutet eine geringere Chance für die Entwicklung einer Angststörung in der Studiengruppe Schwangerschaftsabbruch bis zur SSW 14 + 0 p. m.												
Non-RCT; 1 [8]	Schwerwiegend^e^	Nicht zutreffend^f^	Nicht schwerwiegend	Unentdeckt	Sehr schwerwiegend^g^	Keine	29/149	17/96	18	0,94	−1	Sehr niedrig
										[0,43; 2,03]	[−9; 13]	
**Studiengruppe 1 vs. 3: Schwangerschaftsabbruch bis zur SSW 14** **+** **0 p.** **m. versus Fehlgeburt oder Schwangerschaftsabbruch nach SSW 14** **+** **0 p.** **m. nach initial abgelehntem Wunsch eines Schwangerschaftsabbruchs**												
**Klinisch diagnostizierte Angststörung (von den Frauen berichtet)**												
Interpretation der Effektschätzung: Ein OR größer als 1 bzw. eine positive Effektschätzung bedeutet eine geringere Chance für die Entwicklung einer Angststörung in der Studiengruppe Schwangerschaftsabbruch bis zur SSW 14 + 0 p. m.												
Non-RCT; 1 [8]	Schwerwiegend^e^	Nicht zutreffend^f^	Nicht schwerwiegend	Unentdeckt	Sehr schwerwiegend^g^	Keine	29/149	5/27	19	0,98	−1	Sehr niedrig
										[0,33; 2,93]	[−12; 21]	
**Studiengruppe 1 vs. 4: Schwangerschaftsabbruch bis zur SSW 14** **+** **0 p.** **m. versus Schwangerschaftsabbruch nach SSW 14** **+** **0 p.** **m. (keine Ablehnung des Wunsches nach einem Abbruch)**												
**Klinisch diagnostizierte Angststörung (von den Frauen berichtet)**												
Interpretation der Effektschätzung: Ein OR größer als 1 bzw. eine positive Effektschätzung bedeutet eine geringere Chance für die Entwicklung einer Angststörung in der Studiengruppe Schwangerschaftsabbruch bis zur SSW 14 + 0 p. m.												
Non-RCT; 1 [8]	Schwerwiegend^e^	Nicht zutreffend^f^	Nicht schwerwiegend	Unentdeckt	Schwerwiegend^h^	Keine	29/149	30/228	13	0,70	−4	Sehr niedrig
										[0,44; 1,14]	[−7; 2]	

^a^ Basisrisiko geschätzt durch (medianes) Risiko der Vergleichsgruppen der eingeschlossenen Studie (der Begriff „Risiko“ bezieht sich hier sowohl auf Endpunkte, deren Ereignisse sich für die Frauen negativ, als auch auf solche, deren Ereignisse sich positiv auswirken)

^b^ Unter Qualität der Evidenz wird die studienübergreifende endpunktbezogene Qualität bzw. Vertrauenswürdigkeit der Evidenz verstanden

^c^ Die Bewertung der Studienlimitationen pro Studie pro Endpunkt ist dem Evidenzbericht [1] zu entnehmen

^d^ Adjustiert für Ethnie, Alter, Parität, Bildung, Erwerbstätigkeit, Krankenversicherungsstatus, Drogen und Alkoholprobleme vor der Schwangerschaft, frühere Diagnose einer Depression, früherer Kindesmissbrauch oder Vernachlässigung

^e^ Die Vollständigkeit der Daten (Anteil der Frauen in der Auswertung mit fehlenden Werten zwischen ca. 10–30 %) und die Berücksichtigung prognostisch relevanter Faktoren (fehlende Adjustierung für „frühere Schwangerschaftsabbrüche“) waren nicht adäquat

^f^ Der Faktor inkonsistente (heterogene) Effekte kam für diesen Evidenzbericht nicht zur Anwendung, da jeweils nur 1 Studie pro Endpunkt vorlag

^g^ Das 95 %-KI des OR überdeckt 0,5 und 2. Somit können weder mittelgroße Effekte zuungunsten noch mittelgroße Effekte zugunsten der Studiengruppe 1 (Schwangerschaftsabbruch bis zur SSW 14 + 0 p. m.) ausgeschlossen werden

^h^ Das 95 %-KI des OR überdeckt 0,5 und 1. Somit können weder mittelgroße Effekte zuungunsten noch Effekte zugunsten der Studiengruppe 1 (Schwangerschaftsabbruch bis zur SSW 14 + 0 p. m.) ausgeschlossen werden

*C* kein Schwangerschaftsabbruch bis zur SSW 14 + 0 p. m., *I* Schwangerschaftsabbruch bis zur SSW 14 + 0 p. m., *KI* Konfidenzintervall, *N* Anzahl der Studien, *Non-RCT* nicht randomisierte kontrollierte Studie, *OR* Odds Ratio, *p.* *m.* post menstruationem, *RD* absolute Risikodifferenz, *SSW* Schwangerschaftswoche

Studienlimitationen ergaben sich durch unvollständige Daten zu den berücksichtigten Endpunkten für die beobachteten Studiengruppen. Der Anteil fehlender Daten in den jeweiligen Studiengruppen lag bei ca. 10–30 %, sodass Daten von ca. 70–90 % der Frauen in die Auswertung nach 3 Jahren eingingen. Ein weiterer limitierender Faktor der Studie war die fehlende Berücksichtigung aller wesentlichen Störgrößen bzw. Confounder für das Auftreten späterer psychischer Folgen. Die Ergebnisse wurden zwar für Störgrößen adjustiert, wie z. B. Drogen und Alkoholprobleme vor der Schwangerschaft oder frühere Diagnosen von Depressionen oder Angststörungen, jedoch nicht für wiederholte Schwangerschaftsabbrüche vor Studienbeginn. Diese stellen aus Sicht der Leitliniengruppe eine wesentliche Störgröße dar, weshalb die fehlende Berücksichtigung als weiterer limitierender Faktor der Studienergebnisse beurteilt wurde. Ein weiterer Aspekt betraf die Erhebung der Endpunkte in der Studie. Die klinischen Diagnosen der Depressionen und Angststörungen wurden von den Frauen per Telefoninterview berichtet: Sie wurden gefragt, ob sie eine entsprechende Diagnose von einer medizinischen Fachkraft erhalten haben. Eine Beeinflussung der Ergebnisse aufgrund falscher Angaben ist bei dieser Erhebungsart nicht auszuschließen.

## Einordnung der Evidenzlage

Zur beauftragten Fragestellung der Leitliniengruppe konnte nur sehr wenig Evidenz identifiziert werden. Begründet lag dies vor allem in der fehlenden Übereinstimmung zwischen den in der Fragestellung definierten und den in den ausgeschlossenen Studien berichteten Populationen, Vergleichsgruppen und Endpunkten.

Als relevante Studienpopulation sollten ausschließlich Schwangere mit dem Wunsch nach einem Schwangerschaftsabbruch betrachtet werden. Viele Studien untersuchten ungeplant, ungewollt oder minderjährige Schwangere, jedoch ging aus den Beschreibungen der Studienpopulationen kein konkreter Wunsch nach einem Schwangerschaftsabbruch hervor. Schwangerschaftsabbrüche aufgrund einer medizinischen oder kriminologischen Indikation sollten gemäß der beauftragten Fragestellung ebenfalls nicht eingeschlossen werden. Ein Großteil der identifizierten Studien konnte daher aufgrund der von der Leitliniengruppe definierten Population nicht berücksichtigt werden [1].

Ein weiterer zentraler Grund, der zum Ausschluss von Studien führte, lag in der fehlenden Übereinstimmung der in den Studien beobachteten Gruppen und der in der Fragestellung definierten Studiengruppen begründet. Häufig wurden Frauen, die einen Schwangerschaftsabbruch innerhalb des ersten Trimenons durchführen ließen, und Frauen, die einen späteren Schwangerschaftsabbruch durchführen ließen, gemeinsam in einer Gruppe ausgewertet. Dies konnte zum Teil auf die rechtlichen Gegebenheiten in den Ländern, in denen die Studien durchgeführt wurden und in denen auch spätere Schwangerschaftsabbrüche erlaubt waren, zurückgeführt werden. Da in diesen Studien keine getrennte Darstellung von frühen (innerhalb des ersten Trimenons) und späteren Schwangerschaftsabbrüchen erfolgte, konnten diese Studien ebenfalls nicht berücksichtigt werden. Auch fehlende und unklare Angaben zum Zeitpunkt des Schwangerschaftsabbruchs führten zu einem Studienausschluss [1].

Darüber hinaus stimmte eine Vielzahl der in Studien berichteten Endpunkte nicht mit den Endpunkten überein, welche von der Leitliniengruppe als relevant erachtet wurden. Es sollten ausschließlich klinisch diagnostizierte psychische Erkrankungen oder psychische Störungen untersucht werden. In den meisten publizierten Auswertungen der herangezogenen Turnaway-Studie und weiteren Studien wurden allerdings lediglich Symptomatiken und Risiken für psychische Störungen oder Erkrankungen erhoben und keine Diagnosen für psychische Erkrankungen oder Störungen nach ICD oder DSM [1].

Ein weiterer zu berücksichtigender Aspekt war die aufgrund der definierten Vergleiche fehlende Möglichkeit der Randomisierung der zu beobachtenden Gruppen. Daher wurde bei dieser Fragestellung auf nicht randomisierte vergleichende Studien zurückgegriffen. Da diese per se eine geringere Ergebnissicherheit als randomisierte kontrollierte Studien aufweisen [4], wurde der Einschluss auf prospektiv vergleichende Kohortenstudien mit adäquater Kontrolle der wesentlichen Confounder eingegrenzt, um die Vergleichbarkeit der Studiengruppen zu erhöhen. Die zu beachtenden wesentlichen Störgrößen wurden von der Leitliniengruppe festgelegt. Viele Studien wiesen keine oder keine adäquate Adjustierung der Ergebnisse für diese Störgrößen auf und konnten daher nicht eingeschlossen werden [1].

Nur die eingeschlossene Turnaway-Studie wies eine umfangreiche und adäquate Adjustierung der Ergebnisse auf. Lediglich für frühere Schwangerschaftsabbrüche wurde keine Adjustierung vorgenommen. Dies wurde in der Beurteilung der Vertrauenswürdigkeit der Evidenz als limitierender Faktor berücksichtigt [1].

Ein Überblick über alle ausgeschlossenen Studien mit jeweiligem Ausschlussgrund ist dem Evidenzbericht zu entnehmen [1].

## Ableitung möglicher methodischer Implikationen für das Forschungsfeld

Studien, die sich mit der Bearbeitung der Fragestellung nach psychischen Folgen eines Schwangerschaftsabbruchs im ersten Trimenon im Vergleich zu keinem Schwangerschaftsabbruch im ersten Trimenon befassen, sollten sorgfältig geplant und durchgeführt werden. Aufgrund der Art der Fragestellung und der einhergehenden fehlenden Möglichkeit der Randomisierung ist bei prospektiv vergleichenden Kohortenstudien eine adäquate Confounder-Kontrolle wichtig. Eine systematische Identifikation aller wesentlichen Störgrößen verbunden mit einer adäquaten Adjustierung der Ergebnisse ist erforderlich. Nur so kann eine annähernde Vergleichbarkeit der zu beobachtenden Studiengruppen gewährleistet und eine Verzerrung der Ergebnisse durch Störgrößen reduziert werden [4]. Neben einem höherwertigen Studiendesign und der Rekrutierung einer ausreichenden Anzahl an Studienteilnehmerinnen spielen die Auswahl und die Erhebung der Endpunkte eine wichtige Rolle. Sofern klinisch diagnostizierte psychische Erkrankungen und psychische Störungen untersucht werden sollen, ist eine entsprechende Erhebung durch qualifiziertes Fachpersonal erforderlich. Als weitere Quelle für Studienlimitationen sollte in der Studienplanung ein möglicher Datenverlust aufgrund des Ausscheidens von Studienteilnehmerinnen berücksichtigt werden.

## Fazit

Um eine verlässliche Aussage zur Vertrauenswürdigkeit der Evidenz treffen zu können, sind hohe methodische Anforderungen an Studien zu stellen, die als Evidenzgrundlage für S3-Leitlinien herangezogen werden. Dies gilt auch für die Fragestellung nach psychischen Folgen eines Schwangerschaftsabbruchs im ersten Trimenon im Vergleich zu keinem Schwangerschaftsabbruch im ersten Trimenon.

Die Turnaway-Studie war die einzige Studie, die für den Evidenzbericht des IQWiG zur interessierenden Fragestellung berücksichtigt werden konnte. Sowohl für *klinisch diagnostizierte Depressionen* als auch für *klinisch diagnostizierte Angststörungen* zeigten sich für alle Vergleiche der Studiengruppen bei sehr geringem Vertrauen in die Effektschätzungen keine statistisch signifikanten Unterschiede. Welchen Beitrag die Ergebnisse des Evidenzberichts zum Erstellungsprozess der S3-Leitlinie leisten, entscheidet die verantwortliche Leitliniengruppe.
